# Acute and sub-acute toxicity profile of ultra-hypofractionated low-dose total skin electron beam with two 4 Gy fractions for cutaneous T cell lymphoma

**DOI:** 10.1007/s00432-020-03449-7

**Published:** 2020-11-21

**Authors:** Daniel Rolf, Khaled Elsayad, Hans Theodor Eich

**Affiliations:** Department of Radiation Oncology, University Hospital of Muenster, Building A1, 1 Albert Schweitzer Campus, 48149 Muenster, Germany

**Keywords:** Radiotherapy, Two fractions, Erythema, Bexarotene, Mogamulizumab, Acitretin, Extracorporeal photopheresis (ECP)

## Abstract

**Purpose:**

Low-dose total skin electron beam therapy (TSEBT) over 3 weeks has proved to be a safe and effective treatment for cutaneous T cell lymphomas (CTCL). In this prospective trial, we examined the feasibility of ultra-hypofractionated low-dose TSEBT regimen in two fractions with 4 Gy combined with systemic therapy to minimize the number of visits to radiation centers.

**Patients and methods:**

Six patients with mycosis fungoides (MF) or Sézary syndrome (SS) received TSEBT with a total radiation dose of 8 Gy in two fractions between April 2020 and June 2020. Patient and treatment characteristics, tumor burden, the impact on the quality of life using Skindex-29 questionnaires, and acute toxicities were analyzed.

**Results:**

During TSEBT, all patients developed grade 1 toxicities while two patients developed grade 2 toxicities. One patient experienced sepsis. The most common adverse effects were erythema and edema. All grade 2 toxicities regressed after 4 weeks following TSEBT. Based on the reported symptoms measured by Skindex-29, we detected a significant reduction in total Skindex-29 score after 8 weeks of radiation (*P* = 0.03), particularly in the symptoms (*P* = 0.01) and emotional domains (*P* = 0.04).

**Conclusion:**

Ultra-hypofractionated low-dose TSEBT followed by systemic therapy seems to be a safe and feasible alternative to conventional fractionated TSEBT for patients with MF/SS. The skin tumor burden and the health-related quality of life have been significantly improved within 8 weeks following radiotherapy.

## Introduction

Nowadays, there is a global need to reduce cancer patients and radiation oncology staff’s exposure to the potential risk of COVID-19 infection, or due to limited resources (Chen et al. 2020). Low-dose total skin electron beam therapy (TSEBT) in the range of 10–12 Gy over 3 weeks is a current treatment option for cutaneous T cell lymphoma (CTCL) with excellent response rate and minimal risk of adverse events (Elsayad et al. 2017; Kroeger et al. 2017; Mehta-Shah et al. 2020). Prior studies show that low-dose TSEBT could improve disease symptoms, tumor burden, and patients’ health-related quality of life (HRQL) in CTCL (Elsayad et al. 2020b; Hoppe et al. 2015). TSEBT can be combined with systemic therapy to improve PFS (Elsayad et al. 2020a). During the COVID-19 Pandemic, the International Lymphoma Radiation Oncology Group (ILROG) suggests hypofractionated TSEBT as a valid option for patients with CTCL to reduce the overall treatment duration and exposure to COVID-19 (Yahalom et al. 2020).

Here, we present the feasibility of ultra-hypofractionated low-dose TSEBT followed by maintenance therapies in CTCL.

## Materials and methods

Six hypofractionated low-dose TSEBT courses were administered to six patients with CTCL at our institution during COVID-19 pandemic from 04 to 06/2020 (Table 1). The inclusion criteria involved: patients with a histologically confirmed diagnosis of mycosis fungoides stage IIB–IV or Sézary syndrome and planned TSEBT combined with systemic treatment. The treatment decisions have been taken according to national treatment guidelines, local licensing, and professional information by the treating physicians. Patients have been included after the combination treatment decision has been taken. The study protocol has been presented to the local ethics committee in Münster (Ethik Kommission der Ärztekammer Westfalen-Lippe und Westfälichen Wilhelms-Universität) for approval. Among these patients, four had with mycosis fungoides (MF), and two had with Sézary syndrome (SS). All the patients were clinically symptomatic. Patients treated were asked to complete the Skindex-29 before and after TSEBT (Chren et al. 1996). According to the Common Terminology Criteria for Adverse Events (version 5), treatment toxicities were assessed weekly during treatment periods and at the 4-week follow-up appointment. Skin tumor burden was assessed with the modified skin-weighted severity (mSWAT) score, and pruritus was assessed with a visual analog scale (VAS). All patients were treated with 4 Gy fraction dose over 2 days, to a total dose of 8 Gy using a modified Stanford technique (Page et al. 1970). Supplemental local radiotherapy was delivered to compensate for underdosing in shadowed areas (perineum, axilla, and plantar surfaces) or for large lymph nodes (*n* = 2)/tumorous lesions (*n* = 2).

**Table 1:** Patient characteristics

Patient	Sex/age (years)	Diagnosis/clinical stage	TSEBT dose	Concurrent therapy	Maintenance therapy	Acute toxicities D1–W6	Sub-acute toxicities W8–W12	Response	mSWAT reduction	PFS (W)	FU
1	M/69	SS	2 × 4 Gy	None	Bexarotene	Edema, erythema, and nail atrophy grade 2	Edema and alopecia grade 1	PR	88% at W8	26	Alive without evidence of progression
2	M/66	MF/IIB	2 × 4 Gy	None	Mogamulizumab	Sepsis, alopecia grade 1, and nausea grade 1	Nail changes and alopecia grade 1	PR	87% at W8	26	Died due to COVID-19
3	M/78	MF/IIB	2 × 4 Gy	Bexarotene	Bexarotene	Edema, erythema grade, alopecia, and blistering grade 1	Alopecia grade 1	PR	73% at W8	26	Alive without evidence of progression
4	F/77	SS	2 × 4 Gy	None	Bexarotene, ECP	Erythema grade 2, edema grade 2, alopecia grade 1	Fatigue, nail changes, and alopecia grade 1	PR	63% at W8	22	Alive without evidence of progression
5	M/60	MF/IIB	2 × 4 Gy	Bexarotene	Bexarotene	Erythema and fatigue grade 1	Fatigue, and edema grade 1	PR	89% at W8	19	Alive without evidence of progression
6	F/55	MF/IIB	2 × 4 Gy	Acitretin	Acitretin	Fatigue, erythema, and blistering grade 1	None	CR	100% at W4	18	Alive without evidence of disease

*ECP* extracorporeal photopheresis, *CR* complete response, *W* week, *PR* partial remission, *PFS* progression-free survival, *FU* follow-up, *M* male, *F* female, *MF* mycosis fungoides, *SS* Sézary syndrome

## Results

Characteristics of the patients with CTCL who received low-dose hypofractionated TSEBT at our institution were assessed (Table 1). The median mSWAT score before the start of TSEB for the entire cohort was 52 (range 9–112). Three patients in our cohort received concurrent therapy with retinoid therapy (bexarotene *n* = 3 and acitretin *n* = 1). Furthermore, all patients received maintenance therapy following TSEBT (bexarotene *n* = 4, acitretin *n* = 1, and mogamulizumab *n* = 1). The median follow-up period was 24 weeks (range 18–26). All patients had an objective response with a median mSWAT reduction of 87% (range 63–100, *P* = 0.01). Among four patients with pruritus (median scale: 6, range 2–10), a marked benefit was observed at the follow-up 8 weeks after radiation, with a median score of 2 (range 0–7; *P* = 0.05).

Based on the reported symptoms measured by Skindex-29, the median score before TSEBT was 100 (range 64–130). A significant reduction (*P* = 0.03) in total Skindex-29 score was observed after 8 weeks of radiation with a median score of 78 (range 48–111). In addition, a clinically meaningful difference in the symptoms (*P* = 0.01) and emotional domains (*P* = 0.04) were observed after TSEBT. While, the functioning domains were not significantly improved (*P* = 0.14).

During TSEBT, all the patients experienced mild acute toxicities (Table 1). Four out of six patients developed grade 1 toxicities only while two patients developed grade 2 toxicities. The most common adverse effects were erythema (four patients), followed by edema (three patients). One patient (who had ulcerated lesion) developed sepsis during radiotherapy and was successfully treated with intravenous antibiotics. No patients developed grade 4–5 adverse events. At week 8 of follow-up, all grade 2 adverse events were resolved.

## Discussion

Conventionally fractionated low-dose TSEBT is a safe and effective modality for MF/SS patients, with an ORR reaching 96% and a median interval to the clinical response of 8 weeks (Elsayad et al. 2015; Hoppe et al. 2015; Kamstrup et al. 2015; Morris et al. 2017). Recently, a retrospective study proved the feasibility of hypofractionated TSEBT without moderate or severe toxicities (Jeans et al. 2020). Owing to the potential risk of COVID-19 infection or due to limited resources in various institutions, ultra-hypofractionated TSEBT regimens might be a valid alternative to reduce the number of patients’ visits to radiation centers (Chen et al. 2020). To the best of our knowledge, this prospective clinical trial is the first demonstration of feasibility and toxicity of ultra-hypofractionated low-dose TSEBT in two fractions followed by maintenance systemic treatment for MF/SS patients. Our analysis shows that there are only grades 1 and 2 acute toxicities. Even with concurrent systemic retinoid therapy, there were no grade 4 or higher adverse events.

With RT, clinicians can reduce disease burden and control residual lesions with other topical or systemic treatments (Hoppe et al. 2015). We could show that 8 weeks after ultra-hypofractionated TSEBT, the mSWAT score could be more than halved. Besides, TSEBT is one of the most time-intensive treatments in radiation oncology. With ultra-hypofractionated TSEBT in only two fractions, the exposure of cancer patients and radiation oncology staff to the potential risk of COVID-19 infection can be reduced compared to conventionally fractionated low-dose TSEBT.

Recent studies indicate that maintenance therapy after TSEBT may improve the PFS (Elsayad et al. 2020b; Heumann et al. 2015; Jennings et al. 2019). Low-dose focal radiotherapy combined with romidepsin therapy seems to be safe and reasonable option in patients with symptomatic lesions (Akilov et al. 2012). In the current study, half of the patients (*n* = 3) received concurrent systemic retinoid, and all patients received maintenance treatment without severe toxicities. To enhance the objective treatment response, we evaluated the time to next treatment (TTNT) in our patients. However, TTNT was equal to PFS due to lack of progressions during the follow-up period of 24 weeks (Campbell et al. 2020).

There are several limitations associated with the present study. The number of patients is low, and the median follow-up period is only 24 weeks. Long-term clinical analysis is needed to assess long-term treatment benefits and chronic toxicities.

## Conclusion

Ultra-hypofractionated low-dose TSEBT is a safe and feasible alternative to conventionally fractionated TSEBT for patients with MF/SS to reduce the overall treatment duration and possible exposure to COVID-19 infection. The skin tumor burden and the health-related quality of life were significantly improved following TSEBT.
